# Proceedings of the Æsculapean Society

**Published:** 1850-01

**Authors:** 


### ARTICLE II.

### PROCEEDINGS OF THE ÆSCULAPEAN SOCIETY,

*At its annual session, held at Mount Carmel, Illinois, October 31st*, 1840.

[It affords us much pleasure to lay before our readers the following abstract of the proceedings of this energetic and enterprising association. The paper containing their proceedings came to hand too late for insertion in the proper department, and we therefore insert them here.]

The society met pursuant to adjournment. The President and Vice Presidents being absent, on motion of Dr. J. J. Lescher, Dr. C. J. Miller was called to preside.

The roll being called, Drs. Miller, J. Lescher, J. J. Lescher, J. B. McCarthy, J. M. Logan, and W. B. Norton, answered to the call. The minutes of the previous session, read, and on motion adopted.

Essays being called for, Dr. J. J. Lescher begged leave to read one on Dysmenorrhœ ; in the course of which he endeavored to establish the frequent and annoying complication of painful menstruation with rheumatism, or rheumatic-gout, or in other words, the disease as it manifests itself in a rheumatic, or rheumatic-gouty habit—as is so admirably portrayed by Dr. Rigby.

He also adverted to the magic-like influence of such remedies as Guaiacum, Colchicum, Iod Potass, &c. as is so happily observed in this complication. Upon concluding the essay, it was ordered to be filed.

The Censors being absent, the President appointed the following to serve temporora ly, viz: Drs. J. Lescher, J. J. Lescher, and Norton.

Dr. J. J. Lescher reported a case of cholera-morbus, which was fi ed.

Dr J. J. Lescher, in behalf of the board of censors, reported that Dr. Washburn, of Grayville, had made application for membership, and had upon examination shown himself as properly qualified for admission, and would therefore move that he be admitted; whereupon he was unanimously elected a member.

The Treasurer made his annual report, which was approved.

Dr. J. J. Lescher preferred the following charges and specifications of unprofessional conduct against Dr. David Adams, ex-President of this association, which were ordered to be spread upon the minutes. The motion being made to expel him, the charges were laid over until the next regular meeting, for final action.

"*To the officers and members of the Æsculapean Society.*

GENTLEMEN.—As it is a moral and bounden duty, as well as a high privilege, for each individual member of this association, to watch and foster its interests as an associated body, whose object is to elevate the standard of professional education and intercourse between its members, and to protect every principle calculated to promote its respectability; it is none the less obligatory on us to watch with a jealous eye, every and any violation of its rules of its ettiquettic government, by one of its body.

Such a right being unquestioned, I hesitate the less, inasmuch as I am a junior in this body, to bring to your notice a most flagrant and barefaced violation of one of the fundamental rules of the code of Ethics, as adopted by the society, by one of its members as will appear more plainly in the charges &c., which follow. The character of the charges, gentlemen, as I conceive, are of no trivial nature, but on the contrary, such as demand our most seriousand determined action; calling for such prompt and just men s, as will in future, effectually prevent a similar infringement, alike disgraceful to the offender, and pernicious to this society in its professional bearing. The consequences resulting from his misconduct are calculated to reflect upon every member of this institution, unless we publicly disavow him and his act; for otherwise we become a by-word and reproach in the mouth of every respectable practitioner of medicine, and totally unworthy the name of physicians. In the capacity of President ot this society, he shamefully compels us to endorse an act, for which we entertain the most marked contempt. But to the matter in hand.

Charge 1st.—D . David Adams has been guilty of violating the 14th rule of our code o f ethics

Specification 1st.—The said Dr. Adams did, whilst removing to the South, in the month of January or February last, in the language of the rule referred to, "contribute to the circulation of a secret nostrum," by his act of publicly certifying to the ascribed virtues of a certain Dr. Foot's 'Specific for Cholera and Diarrhœa,' the same certificate appearing in the 'Cairo Delta,' a copy of which is herewith transmitted.

Specification 2nd.---In the certificate aforementioned, he does not alone praise the virtues of the nostrum, but alludes to the high standing and gentlemanly character of the nostrum-monger. He says, "Dr. Foote is a gentleman, and makes no pretensions in which he is not sustained by facts," concluding with the wish that "should his (Dr. F.) lot be cast where the disease (cholera) prevails, his great success on board the steamer Pike No. 8, would justly entitle him to the confidence of the public." In this he is not satisfied with lauding a specific, at best a dangerous resort for so formidable a disease; but to command the greater confidence of the terror-stricken public, he increases the enormity of the offence, by parading the charlatan.

Charge 2nd.—He has, in his vainglorious fondness for p-fessional notoriety, brought into dsrepute the professional dignity of the association, which, by an act of incorporation, should, to say the least of it, command some respect; whose avowed object, amongst others, is to discourage quackery.

Specification 1st.—In the said certificate, he betrayed his assumed official connexion with this society, by which act, we are without our knowledge or consent, drawn into the same silly and gasconading category with himself. Sharing alike with him the just reproach of giving countenance to mountebanks, whose only object is, to rob the credulous and panic-stricken of their money—whose end is, the destruction of human life.

Specification 2nd.---In affixing to his name the title of President, &c., at the time of his certificate, he was guilty of falsehood, of a titular fraud, unbecoming a gentleman and an ex-President of this body; because at the period alluded to, his term of office had expired two years, of which he was not ignorant.

These, gentlemen, are the charges which I feel myself bound to submit to your consideration, trusting, with all due deference, that due justice will be awarded to the offending one. Appealing to your high sense of honor and professional dignity, I entertain not a doubt, but that you will make a deserving and fearless example of the person who dares in his vanity, to degrade his brethren in the sight of strangers; and who is so reckless of truth as to pen to his importance, a filched title.

[Signed,] J. J. LESCHER."

A communication from Dr. George, giving his reasons for absence, was read.

A letter from Dr. E. A. Guilbert, Sec'y of the Med. Chirurg. Society, on the subject of a State Medical Convention, read; and on motion of Dr. J. J. Lescher.

*Resolved*, That this society approve of the project of establishing a State Medical Association; and that this society appoint two delegates to attend the Convention to be held at Springfield, when called.

*Resolved*, That the Secretary enter into correspondence with the Secretary of the Ottawa Med. Chirurg. Society on the subject.

Dr. Miller reported an interesting case of Pleuro-Pneumonia, which elicited much discussion.

Dr. J. J. Lescher gave notice, that he would prefer charges of fraudulent conduct against Dr. Charles H. Harrison.

On motion, the Society adjourned to 8 o'clock, P. M.

WEDNESDAY, 8 o'clock, P. M.

The society met pursuant to adjournment, Dr. Miller in the chair, the roll being called, all present as at the morning's meeting, with the addition of Drs. Sam'l Thompson, James Mahon, and J. Somes.

Dr. J. J. Lescher, in pursuance to notice, read specified charges against Dr. Harrison, an abstract of which was ordered to be spread on the minutes, in words to wit:

[Here follows a series of charges which are of so grave a character that we think best to omit them. If true, they make the accused guilty of a misdemeanor in the eye of the law. They specify a failure to make returns of an agency for periodical publications, which amounts to a breach of trust. But how many men are there in community who are equally guilty of failure to pay over to their creditors, what properly belongs to them, and which, of course, is justly theirs?]

Inasmuch as Dr. Harrison's whereabout was unknown, on motion, the 21st rule of the by-laws was suspended, whereupon the society proceeded to consider the charges, and he was, by a unanimous vote, expelled.

Dr. Thompson made a lengthy and highly edifying report of a case of Intermittent Neuralgia, successfully treated by repeated and large doses of Quinine, conjointly with other judicious remedies.

On motion, the society went into the election of officers for the ensuing year, which resulted as follows:

Dr. Samuel Thompson, President.

Dr. Charles J. Miller, 1st, and Dr. Logan 2nd Vice President.

Dr. W. B. Norton, Secretary.

Dr. J. J. Lescher, Treasurer.

Drs. Banks, Hamilton, and Washburn, Censors.

On motion, Drs. Thompson and J. J. Lescher, were appointed as delegates to attend the State Med. Convention.

A motion to alter the 13th rule of the by-laws, was made and lost,

On motion, the Treasurer was instructed to notify all delinquents of their arrearages, and to urge them to immediate settlement.

The following resolutions were offered by Dr. Logan, which, on motion, were adopted.

*Resolved*, That any member failing to attend for four successive meetings of this association, shall be liable to censure, suspension or expulsion, to be determined by vote of the society.

*Resolved*, That any member being in arrearages to the amount of two dollars and fifty cents, for initiation or certificate fees, assessment or fines, shall be suspended and not allowed to vote or speak in this society, until said arrearages are paid.

On motion, the society adjourned to meet to-morrow morning at 9 o'clock.

THURSDAY, Morning, Nov. 1st, 1849.

The society met pursuant to adjournment, present the same as yesterday, Dr. Thompson in the chair.

On motion of Dr. Washburn, Drs. Thompson, J. J. Lescher, and Logan, were appointed a committee to draft an address, to be sent to each member of this society, one month prior to each meeting. The committee after retiring for a short time, reported a form which was adopted.

Dr. George Lescher's application for membership being reported by the board of Censors; he was unanimously elected a member of this society.

The following appointments were made:

Drs. Mahon and Hamilton, to deliver addresses.

Drs. McCarthy, Washburn, Banks and George, to read essays.

On motion, the Secretary was instructed to furnish a copy of the proceedings of this society to the "Mount Carmel Register" with the request, that the following papers copy, viz:

"The North Western Med. and Surgical Journal," "Vincennes Gazette," "Southern Illinois Advocate," "Olney News," "Lawrenceville Banner," and "Marshal Democrat."

On motion, the society adjourned to meet at Lawrenceville on the last Wednesday in April next.

SAMUEL THOMPSON, President.
W. B. NORTON, Secretary.

